# Proposal of methodology for analysis of stress level based on EEG signals

**DOI:** 10.1186/1753-6561-8-S4-P62

**Published:** 2014-10-01

**Authors:** Alexandre Pomer-Escher, Teodiano Bastos-Filho, Maria Dolores Souza

**Affiliations:** 1 Post-Graduate Program in Biotechnology, Federal University of Espirito Santo, Vitoria, Espirito Santo, 29650000, Brazil; 2 Post-Graduate Program in Electrical Engineering, Federal University of Espirito Santo, Vitória, spirito Santo, 29075910, Brazil; 3 Post-Graduate Program in Psychology, Federal University of Espirito Santo, Vitória, Espirito Santo, 29075910, Brazil

## 

Stress can affect all people, regardless of age, gender or ethnicity. The human body utilizes stress as a response in three different situations, classified according to the way it generates physical, mental or emotional stress. It is important to notice that the nervous system evokes the same physiological responses, no matter what type of stress, and those responses cause a change of level in physical and cognitive performance. This paper presents an analysis methodology of the stress level using brain's signals captured by Electroencephalogram (EEG).

Professionals from Vitoria´s Fire Department, members of *Projeto VIDA *participate as volunteers. The EEG signals are captured through a cap placed over the volunteer's head in order to capture the brain´s signals, using electrodes specifically placed in the frontal cortex at positions Fp1, Fp2, F3 and F4. The results are analyzed together with the peripheral physiological signals, such as: heart beat rate (ECG), peripheral blood flow (at fingers and toes), skin conductance, breath rate and body temperature. A validation study is conducted through a comparison of data available in literature, as well as with evaluation conducted by a psychologist.

The results are analyzed in order to get a correlation between EEG signals and physical mental or emotional stress.
